# Liver enzyme elevation and eosinophilia with atorvastatin: a case of probable DRESS without cutaneous symptoms

**DOI:** 10.1186/s13223-021-00581-y

**Published:** 2021-07-30

**Authors:** Arman Zereshkian, Susan Waserman

**Affiliations:** McMaster University, Hamilton, ON Canada

## Abstract

**Background:**

Drug reaction with eosinophilia and systemic symptoms (DRESS) is a potentially life-threatening hypersensitivity reaction to medication. While a relatively rare phenomenon, early identification and discontinuation of the offending agent is pivotal to patient management. To our knowledge this is the first reported case of probable atorvastatin induced DRESS syndrome without rash.

**Case presentation:**

An adult female presented with 4 days of persistent fevers, abdominal and flank pain, malaise, and generalized muscle weakness without any cutaneous reaction following 20 days of therapy with atorvastatin. She was febrile (38.5 °C), at presentation with a heart rate of 72, and blood pressure of 93/51 mmHg. Her laboratory investigations at their peak demonstrated an Alkaline Phosphatase (ALP) of 792 U/L, Alanine aminotransferase (ALT) of 265 U/L, gamma glutamyl transferase (GGT) of 236 U/L, total bilirubin at 21 mg/dL, eosinophils 3100 cells/µL, leukocytes 20.2 K/µL, hemoglobin of 12.5 gm/dL. During her admission she had normal creatinine and troponin. Her serology for Hepatitis A, B and C were negative. Cytomegalovirus, Epstein-Barr viral serologies were negative. Antinuclear Antibody (ANA), rheumatoid factor, anti-neutrophil cytoplasmic antibody (ANCA), mitochondrial antibody, and smooth muscle antibody were negative. The patient was initially diagnosed as having pyelonephritis due to nonspecific bilateral flank pain but given ongoing fevers and lack of clinical and laboratory improvement with antibiotics, a diagnosis of atorvastatin induced DRESS syndrome was considered probable, and atorvastatin was discontinued. The patients’ clinical status improved gradually without any further therapy and her liver enzymes and eosinophils normalized over the course of a month.

**Conclusion:**

In patients who present with systemic organ involvement and eosinophilia, even without cutaneous manifestations, clinicians should apply the RegiSCAR criteria for DRESS syndrome. This can then help guide treatment with discontinuation of offending agent, or treatment with systemic corticosteroids.

## Background

Drug reaction with eosinophilia and systemic symptoms (DRESS) is a serious, potentially life threatening condition that typically presents with generalized skin rash, fevers, enlarged lymph nodes, hematological abnormalities in addition to eosinophilia and multi-organ dysfunction [1]. The diagnosis of DRESS is often challenging, with heterogeneity in clinical presentation, often initially misdiagnosed as infection [2]. Given this diagnostic dilemma, The Register of Severe Cutaneous Adverse Reactions (RegiSCAR) created a diagnostic validation score which is now widely used for the diagnosis of DRESS syndrome. The criteria are scored from − 4 to 9, with a scores of less than 2 excluding the diagnosis of DRESS, 2–3 being possible, 4–5 probable, and a score above 6 being definite [3].

Many different medications are implicated in DRESS, however the most common causes are typically seen with anticonvulsants and antibiotics [1, 4]. Statin class medications are not typically implicated or associated with DRESS [1]. In our review of the literature, there are 4 case reports of statin associated DRESS syndrome. Given that statins are one of the most commonly used treatments for cholesterol lowering and prevention of cardiovascular disease, further classification of DRESS-like reactions are especially warranted. To our knowledge, we report the first case report of statin associated DRESS syndrome in a patient who did not develop any cutaneous findings during the clinical course.

## Case presentation

A 50-year-old female with a past medical history remarkable for generalized anxiety disorder initially presented to the emergency department with a one-day history of retrosternal chest pain. Her other medications included ASA, Ramipril, and Citalopram. In the emergency department her electrocardiogram (ECG) demonstrated ST segment changes suggestive of a non-ST elevation myocardial infarction (NSTEMI) which prompted urgent coronary artery catheterization. Her coronary angiogram demonstrated Type 3 spontaneous coronary artery dissection (SCAD) with diffuse coronary ectasia and preserved left ventricular ejection fraction. She was treated conservatively with Acetylsalicylic acid 81 mg daily, Ramipril 2.5 mg daily, and Atorvastatin 80 mg daily and discharged two days later.

She presented to the emergency department 20 days later with a 4-day history of fevers (> 38.5 °C) and bilateral flank pain that were non-responsive to non-steroidal anti-inflammatory drugs (NSAIDs). Laboratory investigations in the emergency department were remarkable for leukocytosis (16.6 K/µL), normal eosinophils (300 cells/µL), ALP 365 U/L, ALT 265 U/L, GGT 185 U/L and total bilirubin of 10 mg/dL. Her lipase was normal. Urinalysis in the emergency department demonstrated pyuria and trace ketones without nitrites. She had an unremarkable chest x-ray and computed tomography of the abdomen/pelvis. She was treated with a single dose of Ceftriaxone for presumptive pyelonephritis due to vague complaints of bilateral flank pain and discharged with follow up to her family physician.

Despite this, she continued to have persistent fevers, myalgias, malaise, lower limb weakness, and abdominal pain prompting a return to the emergency department two days later where she was admitted to the hospital with a diagnosis of fever of unknown origin (FUO).

On admission her eosinophils were 500 cell/µL, leukocytes 20.2 K/ µL, ALP 666 U/L, ALT 236 U/L, GGT 236 U/L. Her lipase and urinalysis were normal. Abdominal ultrasound demonstrated borderline gallbladder thickening but no evidence of infection. Blood cultures from two days prior were normal. She had a negative nasopharyngeal swab for Sars-Cov-2 virus. Her vital signs on admission were as follows: Temperature 38.5 °C, heart rate of 72, blood pressure of 93/51, and oxygen saturation of 100 percent on room air. Her physical examination demonstrated a low jugular venous pressure but otherwise normal cardiorespiratory examination. Abdominal examination was normal with a soft, non-tender abdomen and no hepatosplenomegaly. Skin and joint examination were also unremarkable, as were her conjunctivae, and oral mucosa. There was no cervical lymphadenopathy.

On admission she was given a single dose of metronidazole and Ceftriaxone, with her antibiotic regimen subsequently broadened to Piperacillin-Tazobactam. Her serology for Hepatitis A, B and C were negative. Cytomegalovirus, Epstein-Barr virus serologies were negative. Antinuclear Antibody (ANA), rheumatoid factor, anti-neutrophil cytoplasmic antibody (ANCA) as well mitochondrial antibody, smooth muscle antibody were also negative. Gastroenterology was consulted for her elevated liver enzyme tests and abdominal pain. Eosinophils were elevated at 1900 cells/µL, raising the question of a drug reaction causing her hepatitis as opposed to sepsis. Infectious Disease (ID) physician felt that this was drug induced liver injury with eosinophilia and “probable” DRESS syndrome secondary to Atorvastatin as the culprit, given her lack of response to antibiotics and increasing eosinophils.

Ceftriaxone, metronidazole and Atorvastatin were held. Her liver enzymes started to trend downwards, and her symptoms improved. She was discharged in stable condition 2 days later. Eosinophils peaked at 3100 cells/µL prior to discharge. Three weeks into her follow up, eosinophils and liver function test abnormalities had completely normalized. She was asymptomatic one month post discharge and remained off Atorvastatin.

## Discussion

DRESS syndrome is typically a dermatological condition, with a diffuse maculopapular eruptions that progresses to erythematous rash with edema [4]. A retrospective review in one institution in the UK found that nearly all patients diagnosed with DRESS syndrome had cutaneous findings [2]. Our review of the literature revealed four published cases of suspected DRESS syndrome attributed to statin medications, however, all these reported rash as a cardinal finding, unlike our patient. Herein we report the first case of probable DRESS syndrome attributed to a statin without any cutaneous findings.

The diagnosis of DRESS syndrome in this case was made using RegiSCAR diagnostic criteria Table 1 [5]. Our patient scored a 4 indicating “probable” DRESS syndrome. The delay in diagnosis in patients with DRESS syndrome is noted to be on average 1.7 days, with 50% of cases initially being misdiagnosed as infection [2]. In this case, the patient’s diagnosis was reached after 4 days given the lack of classic erythematous rash, possibility of Urinary Tract Infection and later elevation of eosinophils. An important learning point from this case is to consider a diagnosis of DRESS syndrome even in the absence of cutaneous symptoms. Another learning point is the spectrum of hypersensitivity reaction that can occur due to drug therapy. Well described with statins, albeit rare, is drug-induced liver injury [6]. The spectrum of statin hepatotoxicity can be quite variable including ALT elevation and cholestatic liver enzyme elevation, and in a subset of patients, fever and rash [6]. Given the similarity of these findings to DRESS syndrome, this raises the question of whether statin induced hepatotoxicity may be part of the spectrum of DRESS related drug reactions, which requires further study.

**Table 1:** RegiSCAR Criteria for DRESS Syndrome with our patient’s score calculated in the right most column with a total score of 4 (probable)

Scoring Criteria	Score modifier if present	Score modifier if absence	Special score considerations	Scoring for our patient
Fever (≥ 38.5 °C)	0	− 1	–	Present (0)
Lymphadenopathy (> 1 cm in two sites)	1	0	–	Absent (0)
Eosinophilia	1 (If > 700 cells/µL)	0	2 (if > 1500 cells/µL)	Highly present (2)
Atypical lymphocytosis (on blood film)	1	0	N/A	Not assessed (0)
Skin rash (> 50% body surface area)	1	0	–	Absent (0)
Skin Biopsy (if applicable)	1 (if + for DRESS)	− 1 (if negative for DRESS)	–	Not assessed (0)
Organ involvement	1	0	If more than one organ involvement + 1 score for a total of 2	One organ involvement (1)
Resolution > 15 days	0	− 1	–	Present (0)
Other causes excluded (3 or more of below negative) Antinuclear antibody Blood culture Serology for viral hepatitis Chlamydia/mycoplasma	1	0	–	3 causes ruled out (1)

Management of DRESS syndrome is dependent on severity, with mild disease treated with supportive care. Severe disease (such as those with lung or kidney involvement) requires oral glucocorticoids with a slow taper over 6 to 8 weeks. In those who fail to improve with steroids, second line immunosuppressive medications, such as cyclosporine or Intravenous immunoglobulin (IVIG) can be added, although evidence is limited [7].

## Conclusions

DRESS syndrome is associated with significant morbidity and has a 10% mortality rate, hence timely diagnosis is critical [4]. This diagnosis should be considered when patients present with extensive rash with eosinophilia and organ involvement. However, clinicians should remember that rash is not required for a diagnosis of DRESS syndrome and that it should be considered in their differential diagnosis of hypersensitivity drug reactions. While certain medications are more often associated with DRESS syndrome, drug reaction could potentially occur with any medication, and as such, a review of recently started drug therapies is critical.

Statins are a rarely described potential cause of DRESS syndrome, and here we report a case that presented without the typical rash. In patients that present with liver injury after the start of any new medication, diagnostic clarity for DRESS syndrome may be achieved using the RegiSCAR criteria to prevent morbidity and potentially mortality.

## Data Availability

Not applicable.
